# Salivary Biomarkers of Anti-Epileptic Drugs: A Narrative Review

**DOI:** 10.3390/diagnostics13111962

**Published:** 2023-06-04

**Authors:** Ioana-Andreea Chiș, Vlad Andrei, Alexandrina Muntean, Marioara Moldovan, Anca Ștefania Mesaroș, Mircea Cristian Dudescu, Aranka Ilea

**Affiliations:** 1 Department of Oral Rehabilitation, Faculty of Dentistry, University of Medicine and Pharmacy “Iuliu Hațieganu”, 400012 Cluj-Napoca, Romania; chis.ioana.andreea@elearn.umfcluj.ro (I.-A.C.); vlad.andrei@elearn.umfcluj.ro (V.A.); aranka.ilea@umfcluj.ro (A.I.); 2 Department of Paediatric Dentistry, Faculty of Dentistry, University of Medicine and Pharmacy “Iuliu Hațieganu”, 400012 Cluj-Napoca, Romania; 3 Department of Polymer Composites, Institute of Chemistry “Raluca Ripan”, University Babes-Bolyai, 400294 Cluj-Napoca, Romania; mmarioara2004@yahoo.com; 4 Department of Dental Propaedeutics and Aesthetics, University of Medicine and Pharmacy “Iuliu Hațieganu”, 400012 Cluj-Napoca, Romania; mesaros.anca@umfcluj.ro; 5 Department of Mechanical Engineering, Faculty of Automotive, Mechatronics and Mechanical Engineering, Technical University of Cluj-Napoca, 400641 Cluj-Napoca, Romania; mircea.dudescu@rezi.utcluj.ro

**Keywords:** saliva, biomarkers, anti-epileptic drugs, therapeutic drug monitoring, seizure, epilepsy

## Abstract

Saliva is a biofluid that reflects general health and that can be collected in order to evaluate and determine various pathologies and treatments. Biomarker analysis through saliva sampling is an emerging method of accurately screening and diagnosing diseases. Anti-epileptic drugs (AEDs) are prescribed generally in seizure treatment. The dose–response relationship of AEDs is influenced by numerous factors and varies from patient to patient, hence the need for the careful supervision of drug intake. The therapeutic drug monitoring (TDM) of AEDs was traditionally performed through repeated blood withdrawals. Saliva sampling in order to determine and monitor AEDs is a novel, fast, low-cost and non-invasive approach. This narrative review focuses on the characteristics of various AEDs and the possibility of determining active plasma concentrations from saliva samples. Additionally, this study aims to highlight the significant correlations between AED blood, urine and oral fluid levels and the applicability of saliva TDM for AEDs. The study also focuses on emphasizing the applicability of saliva sampling for epileptic patients.

## 1. Introduction

A biomarker can be defined as a “characteristic that is measured and evaluated as an indicator of normal biological processes, pathogenic processes, or responses to an exposure or intervention, including therapeutic interventions” [1,2]. Biomarkers are objective indicators that serve a variety of purposes, from screening and diagnosing conditions to monitoring the effects of treatments or even the progression and prognosis of a disease [1,3].

Most of these biomarkers are assessed through various types of human biological sampling, such as serum, plasma, urine, sputum, etc., depending on the types of investigations required [4]. Blood sampling is usually invasive and anxiety-inducing for patients, with a need for a more restrictive clinical setting. Moreover, there is a limited number of samples that can be collected, and associated difficulties in obtaining those samples from the pediatric and geriatric populations. For that reason, there is a lot of ongoing research regarding the use of other biological matrices for medical investigation purposes, especially for those patients whose clinical status is difficult to assess [1,4].

Saliva biomarker analysis is an emerging field that is attracting an increased interest, being noninvasive and requiring no medical personnel to perform it. Moreover, the fact that it can be performed repeatedly represents the basis of an effective approach in large-scale screening [1]. The hundreds of substances in saliva composition help to detect diseases, provide evidence of exposure to harmful substances and assess overall health status [5].

Saliva composition is influenced by factors such as age, gender, diet, drug intake, level of hygiene, type of stimulus and even the circadian rhythm [5,6,7]. The production of oral fluid can also be quantitatively and qualitatively modified by numerous physiological and pathological conditions [8]. As a result, saliva samples are variable and unstable, with a composition that varies greatly both intra- and inter-individually [5]. Moreover, all its complex biochemical and physical chemical properties make research into saliva more difficult [5]. 

Saliva primarily consists of water (99.5%), along with proteins (0.3%) and inorganic and trace substances (0.2%) [5,7,8]. Glycoproteins, enzymes (e.g., α-amylase), immunoglobulins and antimicrobial peptides are some of the protein constituents, whilst the inorganic component consists of electrolytes (e.g., sodium, potassium, chloride, bicarbonate) [5,9,10].

The drug’s pH, the degree of protein binding, and its molecular weight, spatial configuration and lipid solubility are among the numerous factors that can influence the passage of drugs from blood to saliva [1,8]. Saliva pH values oscillate between 6 and 7, with more alkaline values exhibited when the secretion is increased [5,7]. The blood and oral fluid’s pH influence the passage of drugs from blood to saliva. A more acidic pH of a drug leads to an enhanced drug diffusion. Therefore, acidic drugs are generally present in lower concentrations in oral fluid than blood, whilst alkaline drugs are present at higher concentrations [1,11,12]. On top of that, the substance’s acid dissociation constant (the pK_a_) is a very important factor that determines the potential utility of saliva therapeutic drug monitoring (TDM) for many drugs [1,8]. The pK_a_ is a parameter that characterizes a chemical compound’s ionization equilibria in relation to the compound’s acid–base properties [13]. All the above-mentioned factors influence the passage and, consecutively, the rapport of the blood: saliva drug concentrations.

The objectives of this narrative review are to assess the numerous characteristics of various AEDs and the methods of determining their plasma/serum levels from saliva samples. Additionally, this study aims to highlight the significant correlations between AED blood, urine and oral fluid levels and the applicability of saliva TDM for AEDs. The selected studies focus on AED and TDM from plasma/serum and saliva samples from epileptic patients, from healthy subjects and from in vitro artificially enhanced biofluids. The study also focuses on emphasizing the applicability, the ease and the importance of saliva sampling for epileptic patients.

## 2. Materials and Methods

A thorough electronic literature search was conducted in MEDLINE through PubMed, Web of Science, the Cochrane Library and Google Scholar. The terms used in this process were: anti-epileptic drugs OR anti-epileptics OR acetazolamide OR benzodiazepines OR adinazolam OR alprazolam OR bromazepam OR climazolam OR clobazam OR clonazepam OR clorazepate OR diazepam OR estazolam OR flumazenil OR flunitrazepam OR flurazepam OR halazepam OR loprazolam OR lorazepam OR lormetazepam OR midazolam OR nimetazepam OR nitrazepam OR oxazepam OR prazepam OR temazepam OR triazolam OR brivaracetam OR carbamazepine OR eslicarbazepine acetate OR ethosuximide OR felbamate OR gabapentin OR lacosamide OR lamotrigine OR levetiracetam OR oxcarbazepine OR perampanel OR phenobarbital OR phenytoin OR pregabalin OR primidone OR rufinamide OR topiramate OR valproic acid AND saliva OR oral fluid OR salivary biomarker.

The inclusion criteria were as follows: any study that described the determination of any of the aforementioned AEDs through sampled or enhanced oral fluid. Commentaries, opinion articles, editorials and conference abstracts were excluded.

After removing duplicates, the titles and abstracts of the articles were read and then, if the studies fit the criteria, the full text was examined and a decision was made regarding study inclusion in this review.

## 3. Anti-Epileptic Drugs

Anti-epileptic drugs (AEDs) are structurally and functionally diverse drugs prescribed in a number of conditions such as epilepsy, neuropathic pain, mania, anxiety or spasticity. AEDs have clinically relevant differences, leaving the choice of the prescribed drug to be purely empirical [14].

TDM has the optimization of a patient’s clinical outcome as an objective, identifying the initial response to a medication and the need for any adjustments [15,16]. TDM supports the management of patients’ medication regimens with the aid of measured drug concentrations [15].

Predicting an optimum dose of an AED for a particular patient is an impossible task. Although there are well-defined reference ranges established for most AEDs, the individual differences and the severity of epilepsy make it impossible to accurately pinpoint an optimal dosage [15,16]. In some patients, dosages below the target range can manage seizures well, whilst other patients can require and tolerate drug concentrations in excess of the range [17]. Moreover, seizures occur at irregular intervals, with the clinical symptoms of epilepsy and the signs of toxicity not always being detectable. Since anticonvulsivant therapy is long term, determining if and what AEDs are causing more harm than good is essential. All these aspects, plus the fact that there are no direct laboratory markers for clinical efficacy or for drug toxicity, make it difficult to ascertain if a prescribed dose will be sufficient to control seizures in the long term. Since the correlation between AED serum concentrations and the clinical effects is superior to the correlation between dose and effect, measuring drug concentrations is often the most effective way to guide treatment [15,16].

For most AEDs, saliva reflects the free (pharmacologically active) serum concentrations [15]. Only the fraction of the drug that is unbound from serum proteins is available to diffuse from the vascular system and accumulate in tissues, and to be available for interaction with therapeutic targets. Therefore, the extent of serum binding can have significant effects on the pharmacodynamic properties of a compound, as well. It is, however, important to wait for the drug to reach an equilibrium between the saliva and the blood levels. This equilibrium varies from one person to another and from one drug to another [15,16]. Aman et al. suggest performing regular salivary TDM correlated with neurological assessments, in order to avoid toxic drug concentrations [18].

There are, however, factors that influence the interpretation of the saliva sample, such as the patient’s use of concomitant drugs, how the sample was collected, stored and/or analyzed, and the timing of the sample collection in relation to the last orally administered dose [1,16].

Anti-epileptic medication should ideally be introduced slowly, with doses gradually increasing depending on symptoms. The AED should be titrated upwards to the maximum tolerated dose only if seizures still continue to occur. Any type of change in therapy should be made one at a time, gradually, in order to avoid toxicity. Saliva TDM can help with any dose increase in order to predict/avoid toxicity, as side effects are often insidious and might go unrecognized. If the patient has no benefit from a maximum tolerated dose of a drug, the treatment should be switched to an alternative first-line drug [19]. When switching medications (even though the drugs are considered bio-equivalent to the branded product), there may be differences in the drug’s bioavailability and, therefore, in the clinical status of the patient, causing potential breakthrough seizures. The determination of AED concentrations is a good practice before and after switching a patient’s medication, in order to ascertain both drugs’ bioavailability [17].

In clinical practice, about 30% of patients are pharmacoresistant, which can cause high rates of disability, morbidity or mortality [20,21]. During epileptic seizures, using saliva monitoring, drug oscillations can be assessed and concentrations can be correlated with therapeutic profiles, thus avoiding toxicity [20].

In children, dose requirements are less predictable than for adults, being constantly subjected to change—in these cases, TDM is a must for patient management. Since plasma sampling may present difficulties in children, saliva TDM can be particularly helpful. In pregnant women, due to all the metabolic changes, the pharmacokinetics of many AEDs are altered, causing plasma concentrations to more or less significantly decline. Saliva samples reflect the non-protein-bound quantity of AED in plasma, therefore making frequent TDM in pregnant women easier. In the elderly, the plasma concentration is affected by greater pharmacodynamic sensitivity, thus complicating the interpretation of TDM results. Moreover, drug polytherapy is significantly increased in the elderly compared to other age groups, and drug interactions are more likely to occur. Therefore, saliva monitoring fulfills the need for a rapid method for TDM [17].

When it comes to patients with co-morbidities and co-pathologies, possible drug–drug interactions can result in either an increase or a decrease in plasma AED concentrations. Moreover, the absorption, distribution, elimination and protein binding of AEDs can be seriously affected, resulting in either signs of toxicity or with patient experiencing breakthrough seizures. Therefore, the rapid and correct measurement of AED concentrations is essential [22]. In patients with hepatic disease, the elimination of AEDs can be significantly altered, and therefore the prediction of the extent of change in AED clearance can be impossible. Consequently, in these situations, TDM is essential and considered the best practice [17].

## 4. Salivary Levels of Individual AEDs

### 4.1. Acetazolamide

Acetazolamide has a pK_a_ of 7.2, with approximately half of its molecules being charged in blood and oral fluid at a physiological pH. It has a plasma protein binding of 95%, which predicts a poor penetration into oral fluid [16].

Acetazolamide is a drug with no clinically important drug–drug interactions, with a predictable dose-concentration relationship that does not recommend routine TDM [16].

However, two studies by Wallace et al. and Hartley et al. reported saliva as an appropriate source for acetazolamide TDM. The studies were, however, performed on healthy volunteers and the samples were collected within the first hour of administration, which might influence the oral fluid concentrations. A correlation coefficient of r^2^ = 0.99 and recoveries of more than 87.7% up until 100% were reported [23,24].

### 4.2. Benzodiazepines

Benzodiazepines act as neural inhibitors, resulting in a slowing of neurotransmission. Commonly used to prevent seizures and to treat anxiety and sleep disorders, their main effects include sedation, hypnosis, tranquilization, decreased anxiety, centrally mediated muscle relaxation and anti-convulsant activity. Among the common side effects, the significant impairment of mental alertness and cognitive performance, as well as amnestic effects, are probably the most notable [25,26,27].

Some of the most well-known and frequently prescribed benzodiazepines are adinazolam, alprazolam, bromazepam, climazolam, clobazam, clonazepam, clorazepate, diazepam, estazolam, flumazenil, flunitrazepam, flurazepam, halazepam, loprazolam, lorazepam, lormetazepam, midazolam, nimetazepam, nitrazepam, oxazepam, prazepam, temazepam and triazolam [25,26].

Benzodiazepines bind to plasma proteins, having low pK_a_ values. For that reason, they are generally found in low concentrations in saliva samples, showing a shorter detection time than in blood [28]. Benzodiazepines are known to bind the protein albumin, but mainly on α-glycoprotein. Therefore, due to their consequent low concentration in biofluids, high sensitivity is required for the determination of benzodiazepines in biological samples [27].

Clobazam is prescribed for the treatment of various epilepsies (in generalized seizures, for the adjunctive intermittent treatment of partial seizures and for the management of the non-convulsive status epilepticus) and febrile and alcohol withdrawal seizures [17]. Clobazam and its pharmacologically active metabolite, N-desmethyl clobazam, have a plasma protein binding of 85–90%. The metabolite is present in blood at much higher concentrations than the parent drug [16,17]. One study by Bakke et al. used cut-off limits primarily selected based on the sensitivities of the used analytical methods [29]. There have been reports of clobazam’s excessive accumulation correlated with toxicity in patients. Nevertheless, clobazam and N-desmethyl clobazam can be monitored in saliva samples—moreover, the salivary concentrations are highly correlated with serum concentrations (r^2^ = 0.93 and r^2^ = 0.90) [15,16,17,30,31].

Clonazepam is used for the treatment of various seizure types, in Lennox–Gastaut syndrome and in the management of status epilepticus. It is as yet unknown whether clonazepam is secreted into saliva [16]. The elimination of clonazepam is associated with individual differences and variability in the dose-to-plasma concentration relationship [17]. Hart et al. analyzed saliva samples spiked with clonazepam—the samples that were stored overnight at room temperature had drug concentrations 76% lower compared to samples that were analyzed immediately. These findings suggest the fact that clonazepam is unstable in saliva [32]. Moore et al. reported a correlation coefficient of r^2^ = 0.9991 for clonazepam, after oral fluid was fortified with several benzodiazepines at the concentration of 10 ng/mL [33]. Bakke et al. reported that clonazepam is part of the benzodiazepines he found to be less detected in oral fluid compared to blood [29]. Desharnais et al., in a recent study published in 2020, used a Quantisal^®^ device to collect saliva samples and, using incubation with a precipitation solvent, determined 7-aminoclonazepam in oral fluid samples, but without quantifying its concentration. The authors stated a recovery < 80% for 55 out of the 97 analyzed compounds [34]. Using HPLC, Uddin et al. reported a correlation coefficient of r^2^ = 0.999 for clonazepam in saliva samples [27]. Using an LC-MS/MS method, Concheiro et al. also reported a correlation coefficient of above 0.99 for several tested drugs (including benzodiazepines and, consequently, clonazepam) [35]. Øiestad et al., using the same method, reported a correlation coefficient of r^2^ = 0.993 for clonazepam [36]. Using long-column fast gas chromatography/electron impact mass spectrometry (GC/EI-MS), Gunnar et al. quantitated 30 different drugs of abuse from 250 μL of oral fluid, thus determining clonazepam with a 72.8% recovery and with a 0.992 correlation coefficient [37]. 

Diazepam, while being licensed as a skeletal muscle relaxant, an anxiolytic and a sedative and analgesic, is targeted for the management of febrile convulsions and of status epilepticus. Diazepam is metabolized in the liver to its pharmacologically active metabolite, N-desmethyldiazepam (nordiazepam), with both further metabolized to temazepam and, respectively, oxazepam. N-desmethyldiazepam accumulates in plasma to higher concentrations than diazepam, being responsible for most of the clinical effect. Many patients tend to develop tolerance to the anti-seizure effects of diazepam. Therefore, there are differences between patients when it comes to the dose-to-plasma concentration relationship, as well as the plasma concentration to clinical effect relationship. Both diazepam and N-desmethyldiazepam distribute into saliva, the concentrations reflecting their non-protein bound plasma concentration [17]. Hallstrom et al., in a study published in 1980, reported a correlation coefficient of r^2^ = 0.89 between salivary and plasma diazepam and r^2^ = 0.81 between salivary and plasma nordiazepam [38]. Moore et al. reported a correlation coefficient of r^2^ = 0.9996 for diazepam in oral fluid samples [33]. Gunnar et al. reported a correlation coefficient of r^2^ = 1.000 for diazepam, with a 63.3% recovery [37]. Bakke et al. reported that diazepam was more often detected in blood samples than in oral fluid [29]. Vindenes et al. stated that benzodiazepines were most commonly detected in urine rather than oral fluid, but, however, N-desmethyldiazepam was substantially more detected in oral fluid samples, with a sensitivity of 95%. This study mentioned cut-off values for all of the screened and confirmed 32 most commonly abused drugs [22]. Gjerde et al. reported correlation coefficients of r^2^ = 0.61 for diazepam and r^2^ = 0.95 for nordiazepam, with low oral fluid/blood ratios of 0.036 for diazepam, and, respectively, 0.027 for nordiazepam. This study also mentioned cut-off concentrations for all the 17 tested drugs [39]. Christodoulides et al., using a chip-based Programmable Bio-Nano-Chip platform and LC-MS/MS, detected diazepam from oral fluid samples in approximately 10 min [40].

Midazolam is a short-acting benzodiazepine prescribed as a hypnotic, anesthetic or for the treatment of status epilepticus or generalized seizures. Link et al. reported in their study a liquid chromatography/electrospray ionization tandem mass spectrometry method that was successfully applied to midazolam and its metabolites (1-hydroxymidazolam and 4-hydroxymidazolam). In both oral fluid and plasma, the method showed a good sensitivity in determining midazolam and its metabolites [41]. In another ulteriorly published study, Link et al. noted that the concentrations of midazolam and its metabolites were much lower in saliva than in plasma, although there was a significant linear correlation between midazolam levels in both matrices. The authors also concluded that oral fluid sampling is a good way of determining midazolam and its hydroxy-metabolites, although, because of their low concentrations, sensitive methods are to be used [42]. Using a triple quadrupole LC-MS-MS system, Moore et al. reported a mean recovery of 81.48% of midazolam from oral fluid samples [32]. Using long-column fast gas chromatography/electron impact mass spectrometry (GC/EI-MS), Gunnar et al. determined midazolam with a 73.1% recovery and with an r^2^ = 0.997 correlation coefficient by using CG/EI-MS [37]. Donzelli et al. simultaneously determined six probe drugs through phenotyping CYP isoforms (human cytochrome P450 enzymes) [43]. These isoforms are involved in the metabolism of many xenobiotics and are responsible for the oxidative metabolism of approximately 50–90% of commonly used drugs [43,44]. The authors have concluded that for midazolam, when a higher dose of 7.5 mg is administered, saliva has a usefulness for non-invasive phenotyping of CYP3A4. Moreover, due to its short plasma half-life, midazolam cannot be reliably determined at timepoints later than 4 h [43]. 

Benzodiazepines are detectable in oral fluid, but, for the most part, at lower concentrations in urine [45,46]. Moore et al. used the Quantisal^®^ collection device, quantified using solid-phase extraction for analyzing benzodiazepines in oral fluid, and detected them with the use of liquid chromatography with tandem mass spectrometric detection. The authors simultaneously quantified a total of 14 benzodiazepines, with a percentage recovery from 81.4% (the lowest) to 90.17% (the highest), reporting intraday precision assays of 2.8–7.29% [32]. Desharnais et al. also used the Quantisal^®^ collection device. Samples were prepared with an organic precipitation solvent in order to boost drug recovery and the stability of benzodiazepines, and then analyzed with LC-MS/MS [34]. Valen et al. used Intercept^®^ oral fluid sampling kits, but admitted that better recoveries and fewer matrix effects were observed for some substances when Quantisal^®^ kits were used. The authors reported extraction recoveries between 58% and 76% for most tested drugs and recoveries between 23% and 33% for three 7-amino benzodiazepines metabolites [47]. Uddin et al. developed an HPLC method with diode array detection (DAD), in order to determine six benzodiazepines and two metabolites in plasma, urine and saliva samples. The mean recoveries reported for plasma, urine and saliva were 96.0–108.2%, 94.3–107.1% and 97.0–107.0% in within-day assays [27]. Bakke et al. reported, using ultra high-performance liquid chromatography-tandem mass spectrometry (UHPLC-MS-MS) on blood and oral fluid samples, that oxazepam was detected more frequently in oral fluid compared to blood (100% versus 34%). Alprazolam and nitrazepam were detected more frequently in blood compared to oral fluid (100% compared to 69.1% and, respectively, 93.5% compared to 51.6%) [28]. Pil and Verstraete reported that during the “Rosita 2” study, where 10 devices for roadside drug testing for oral fluid were evaluated and over 2000 tests were performed on over 2000 people, sensitivity for benzodiazepines varied between 33% and 69%. For benzodiazepines, in oral fluid samples, the mean sensitivity, specificity and accuracy were reportedly 74.4%, 84.2% and 79.2%, while for whole blood samples these mean percentages were 66.7%, 87.0% and, respectively, 74.4% [48]. Inscore et al., in their published study, developed a new patented method that allowed the detection of five different drugs at 1 ppm in oral fluid in less than 10 min. The method used surface-enhanced Raman spectroscopy (SERS), using gold- and silver-doped sol-gels immobilized in the glass capillaries. The electronegative gold and the electropositive silver’s purpose was to attract differently charged chemical groups [49].

### 4.3. Brivaracetam

Brivaracetam is a novel member of the racetam family of anticonvulsants, prescribed as an adjunctive therapy in partial-onset seizures of epileptic patients [17,50]. It has a wide interindividual variability in the rate of elimination, having a weak plasma protein binding of 35% [17,51]. Brivaracetam plasma concentrations decrease when carbamazepine, phenobarbital, rifampin or phenytoin are administered. Therefore, monitoring plasma concentrations is indicated for evaluating a possible toxicity and for ascertaining possible clinical interactions [17]. Brivaracetam is a small, non-ionizable molecule, with a diffusing capacity from plasma to saliva. It distributes into saliva, the concentrations reflecting the non-protein bound concentration in plasma [17,50].

Rolan et al. reported that oral fluid is a suitable analytic matrix for brivaracetam, with the saliva concentrations being highly correlated to plasma concentrations (r^2^ = 0.97), with a slope (standard error) similar to the protein-unbound fraction of brivaracetam [50].

### 4.4. Carbamazepine

Carbamazepine is a first-line drug in the treatment of partial and primary or secondarily generalized seizures. It is also prescribed for treating trigeminal neuralgia and bipolar disorder. Its pharmacologically active metabolite is carbamazepine-epoxide, which accumulates in plasma [17].

Carbamazepine metabolism can be affected by numerous AEDs to increase its blood concentration (clobazam, stiripentol) or decrease it (felbamate, oxcarbazepine, phenobarbital, phenytoin, primidone, rufinamide) [17,52]. Simultaneously taking carbamazepine and lamotrigine may increase the prospect of neurotoxic side effects [53]. Carbamazepine metabolism can also be affected by many non-epilepsy drugs to increase its blood concentration (such as clarithromycin, ciprofloxacin, erythromycin, fluconazole, metronidazole, miconazole, etc.) or decrease it (such as rifampicin, risperidone, etc.). Other drugs can increase carbamazepine–epoxide concentrations and may cause toxicity, such as brivaracetam, valproic acid, zonisamide, etc. [17,52]. 

Although it is stated that saliva stimulation before probing can affect the drug’s pH and, therefore, its determined concentration, for carbamazepine it seems that salivary stimulation, the pH of saliva or the volume of fluid produced have no influence on its determined concentration. Stimulating salivation, besides enabling sampling in dehydrated or comatose patients, does not alter carbamazepine concentrations in saliva [36,37,54,55,56,57]. Carbamazepine and carbamazepine–epoxide are 70–75% and, respectively, 50–60% bound to plasma proteins [16,17,51,58]. Therefore, the salivary concentration of both substances is similar to the free concentration of the pharmacologically active, non-protein bound concentration in plasma [15,17]. Usually, carbamazepine-10,11-epoxide is at a steady state of 15–20% of the total carbamazepine concentration for most patients [59]. Considering patient inter-individuality and many drug-to-drug interactions, the TDM of carbamazepine and its metabolite is essential in order to ensure an optimal therapeutic response and to avoid toxicity [17]. Its narrow effective range requires constant monitoring, with repeated blood draws from patients. Therefore, saliva TDM is proposed to non-invasively assess and monitor carbamazepine concentrations [60]. Vasudev et al. reported that the measurement of the unbound concentration of carbamazepine from saliva should induce a better correlation with seizure control [58].

Patrick et al., in their review article, concurred that carbamazepine and carbamazepine–epoxide concentrations in saliva correlate with concentrations in total serum (r^2^ = 0.84–0.99 and, respectively, r^2^ = 0.76–0.88) [16]. Dordević et al. used HPLC with UV detection in order to determine carbamazepine from both serum and saliva samples. The authors noted a strong correlation between the two matrices (r^2^ = 0.9481) [53]. Vasudev et al. studied saliva and blood samples that were centrifuged and analyzed using HPLC. The authors expressed a good linear relationship between the samples from the two matrices, with a correlation coefficient of r^2^ = 0.659 [58]. Al Za’abi et al. simultaneously quantified carbamazepine in saliva and serum samples using a fluorescence polarization immunoassay with a TDx analyzer. The authors reported a good linear relationship (r^2^ = 0.99) between the saliva and serum samples, with a 1.02 ± 0.11 mean ratio of carbamazepine salivary to serum-free concentration [61]. Djordjević et al., also using HPLC-UV, analyzed carbamazepine saliva and serum levels in healthy and in acutely poisoned patients. The authors reported lower carbamazepine concentrations in saliva with regard to serum levels when samples from the two matrices were collected at the same time. In patients with acute poisonings, consequent to different ingested doses of carbamazepine, the authors noted high inter-individual variations, with a strong correlation between saliva and serum levels (r^2^ = 0.9117). In poisonings, due to a saturation of finding proteins and an increase in free serum carbamazepine levels, they also reported an average higher ratio of saliva and serum (0.43) than in the long-term use of therapeutic doses (0.39) [55]. Carona et al., using a novel HPLC technique with DAD, reported a plasma and saliva correlation of r^2^ = 0.8299 for carbamazepine and of r^2^ = 0.9291 for carbamazepine-10,11-epoxide [20]. Dziurkowska and Wesolowski, using UHPLC with a DAD, successfully detected carbamazepine and carbamazepine-10,11-epoxide from saliva samples. The method used was reported to have good linearity, reflected by r^2^ > 0.99 for all the analyzed substances [62]. Carvalho et al. also reported using LC coupled to a diode detector in order to determine carbamazepine and other AEDs from oral fluid, although they determined the drugs from dried saliva spots. A mean recovery for carbamazepine was reported between 40.8 and 45.5%. The authors adapted cards that are commonly applied in dried blood spots sampling to oral fluid sampling and reported a linearity between 0.1 and 10 μg/mL for all AEDs [59]. Dwivedi et al. noted the statistically significant association of carbamazepine levels in serum and saliva, also reporting a positive correlation between the carbamazepine daily dose and the plasma levels [63]. Dziurkowska and Wesolowski tested deproteinization with 1% formic acid solution in acetonitrile, in order to determine carbamazepine and its metabolite from oral fluid. The authors reported a good linearity in the concentration range of 10–5000 ng/mL (r^2^ > 0.999) and an extraction recovery of over 95% [64]. Chen et al. proposed using SERS as a faster method, which was non-contact, label-free and economic, and does not require professionals in order to determine on-site carbamazepine in oral fluid. The method was based on Au-Ag core–shell nanomaterial substrates that greatly improved the signal of the target molecule and, consequently, increased the detection sensitivity [60]. Capule et al. studied the connection between carbamazepine treatment and Stevens-Johnson syndrome/toxic epidermal necrolysis (SJS/TEN). The authors extracted and analyzed genomic DNA from saliva samples, using a UV–visible spectrophotometer and then genotyping HLA-A alleles by polymerase chain reaction. Despite their small sample size, a significant correlation between HLA-B75 and HLA-B*15:02 alleles and carbamazepine-induced SJS/TEN was reported [65]. Therefore, based on all the aforementioned studies, saliva is a good matrix for carbamazepine TDM.

### 4.5. Eslicarbazepine Acetate

Eslicarbazepine acetate is a licensed AED used in the adjunctive treatment of partial onset seizures. Its non-licensed uses include the treatment of bipolar disorder, cranial or trigeminal neuralgia, headache and neuropathic pain [17].

Carbamazepine, phenytoin and topiramate enhance eslicarbazepine acetate’s elimination and, therefore, decrease its plasma concentrations [17,52]. Eslicarbazepine has linear pharmacokinetics, with protein binding of 30% [16]. Its pharmacologically active metabolite is eslicarbazepine, similar to oxcarbazepine’s active metabolite, 10-hydroxycarbazepine, which is secreted into saliva, having a good correlation with plasma levels [15,17]. There were no studies found that quantify eslicarbazepine acetate from saliva samples. Patrick et al. stated in their 2013 review paper that, since eslicarbazepine is the same molecule as 10-hydroxycarbazepine, it can be expected that its saliva transfer will be similar [16].

### 4.6. Ethosuximide

Ethosuximide is an AED that is prescribed in the monotherapy of absence seizures [17]. It is not protein-bound, and therefore it is distributed into saliva at similar concentrations in plasma, with correlations between the ethosuximide levels of the two matrices (r^2^ = 0.99). Therefore, for this drug, saliva TDM can be performed [16,17].

### 4.7. Felbamate

Felbamate is an AED that is prescribed to patients with Lennox–Gastaut syndrome and to those who do not respond well to alternative treatments. Due to the formation of a reactive atropaldehyde metabolite which can cause toxicity in some individuals, felbamate has been correlated with an increased risk of aplastic anemia and hepatotoxicity, which lead to the restriction of its use [16,17]. Carbamazepine and phenytoin enhance felbamate’s elimination, decreasing its plasma concentrations. Valproic acid inhibits felbamate’s metabolism and gabapentin inhibits felbamate’s renal elimination, thus both increasing felbamate’s plasma concentration. Felbamate is 48% bound to plasma proteins [17]. To date, saliva TDM for felbamate is still unstudied [16,17].

### 4.8. Gabapentin

Gabapentin is prescribed in the monotherapy treatment of partial seizures and peripheral neuropathic pain, and as an adjunctive treatment in the epilepsy of adults and children over 6 years of age [16,17]. Gabapentin is not protein bound and not metabolized, its clearance being entirely performed by renal excretion [16,17,66]. Gabapentin is reported to not interact pharmacokinetically with other AED, nor to alter their serum levels [66]. Since gabapentin is not protein bound, salivary levels are assumed to be similar to those in serum [16,66]. Studies have shown that gabapentin is secreted into saliva at lower concentrations than it is found in plasma, but nonetheless with a significant correlation between its levels in the two matrices [16,17,66]. Pujadas et al. successfully determined gabapentin levels in oral fluid using GC-MS and a solid-phase extraction procedure. However, the reported recovery values were 8.2%, 8.8% and 19.7% [54]. Berry et al., using reversed phase HPLC, reported that 5–10% of serum gabapentin concentrations were found in saliva samples, possibly relating that fact to its hydrophilic character. The authors noted that, while there is a linear relationship between gabapentin salivary levels and dosage increments, the saliva TDM of gabapentin is more a means to confirm that the patient has taken the drug rather than for quantifying it for therapeutic monitoring [66]. 

### 4.9. Lacosamide

Lacosamide is prescribed in the mono- and adjunctive therapy of partial onset seizures in epilepsy [16,17]. Enzyme-inducing AEDs such as carbamazepine, phenytoin and phenobarbital can decrease plasma lacosamide concentrations by enhancing its elimination [17].

Lacosamide’s binding to plasma protein is 14%, with its saliva concentrations reflecting the non-protein bound plasma concentrations [17]. While Carona et al. used the HPLC method to determine AEDs in saliva, they reported a mean recovery of lacosamide of 86.6% ± 7.33 of saliva samples [20]. Greenaway et al. reported, in their study, a correlation coefficient of lacosamide levels between serum free concentrations and saliva concentrations of r^2^ = 0.828, while Brandt et al. reported a coefficient interval of r^2^ = 0.842 [67,68]. Cawello et al. reported a ≤10% difference for saliva and total plasma lacosamide concentration ratio [69]. Patrick et al., in their review article, reported a mean saliva/serum lacosamide concentration coefficient interval of r^2^ = 0.84–0.98, all these findings proving that saliva is a suitable source for investigating lacosamide pharmacokinetics [16].

### 4.10. Lamotrigine

Lamotrigine is prescribed for the monotherapy treatment of partial and generalized tonic–clonic seizures and also as an adjunctive treatment in seizures and Lennox–Gastaut syndrome. It has other uses in bipolar depression, migraines, neuropathic pain, peripheral neuropathy, psychosis, schizophrenia and trigeminal neuralgia [16,17]. Some of the AEDs that inhibit lamotrigine metabolism and increase its concentrations are felbamate and valproic acid, while carbamazepine, eslicarbazepine acetate, methsuximide, oxcarbazepine, phenobarbital, phenytoin, primidone, retigabine and rufinamide decrease lamotrigine concentrations by inducing its metabolism. Other non-AED pharmacokinetic interactions of lamotrigine include aripiprazole, isoniazid and sertraline, which increase its concentrations, and, respectively, acetaminophen, atazanavir, ethambutol, olanzapine, oral contraceptives, rifampicin and ritonavir, that decrease its blood concentrations [17,52]. Pregnancy can also reduce lamotrigine concentrations [70].

The fact that there are numerous drug–drug pharmacokinetic interactions and, also, that there are large inter-individual differences in dose-to-plasma concentrations, make lamotrigine TDM valuable and necessary [17]. Lamotrigine is 55–66% bound to plasma proteins, with its saliva concentrations reflecting the non-protein bound plasma levels [16,17,51].

Patrick et al. noted that earlier studies reported a high correlation between saliva and serum concentrations of lamotrigine (r^2^ = 0.95) [16]. Tsiropoulos et al. studied the correlation between lamotrigine concentrations in serum and saliva, while also examining the relationship between the saliva levels and the non-protein bound lamotrigine concentrations in serum. Both stimulated and unstimulated saliva from the same patients were tested, demonstrating a good correlation between lamotrigine serum concentration in both cases (unstimulated and stimulated: r^2^ = 0.85 and r^2^ = 0.94, respectively). Saliva lamotrigine concentrations were reported to be in good correlation with the free, non-bound levels [71]. Malone et al. also studied lamotrigine concentrations in both stimulated and unstimulated saliva samples, comparing them to serum samples. The authors reported a mean saliva/serum lamotrigine concentration ratio of 0.49 at a serum lamotrigine concentration of 10 mg/L, with a correlation coefficient of r^2^ = 0.9841. The authors concluded that, with appropriate timing in sampling, saliva could provide a good alternative for lamotrigine TDM [72]. Incecayir et al. also reported a good lamotrigine saliva/serum correlation of r^2^ = 0.677, while Mallayasamy et al. and Kuczynska et al. reported values of r^2^ = 0.683 and, respectively, r^2^ = 0.82 [70,73,74]. Ryan et al. reported good lamotrigine salivary and serum concentrations (r^2^ = 0.81–0.84) in both patients under 16 years of age and also adults [75]. Conclusively, saliva TDM is a viable option in monitoring lamotrigine levels. 

### 4.11. Levetiracetam

Levetiracetam is prescribed in the monotherapy treatment of partial seizures, as well as for adjunctive therapy and in primary generalized tonic–clonic seizures associated with idiopathic generalized epilepsy and myoclonic seizures. Carbamazepine, lamotrigine, methsuximide, oxcarbazepine, phenobarbital and phenytoin can lower levetiracetam plasma concentrations by enhancing its metabolism [16,17].

Levetiracetam has an oral bioavailability of 100%, is 3–10% protein bound and is secreted into saliva, the concentrations being highly correlated with those in plasma [16,17,76,77].

Lins et al., in their study, have reported that when performing oral fluid TDM for levetiracetam, the last oral dose is important because administration within two hours of saliva sampling leads to high drug concentrations. The authors recommend saliva TDM for levetiracetam to be performed at least four hours after oral intake [16,78]. Moreover, Grim et al. noted that stimulated saliva samples can result in lower concentration values (r^2^ = 0.87 stimulated saliva, whereas r^2^ = 0.91 in unstimulated samples) [76]. Several studies noted a saliva/serum ratio of almost 1/1, matching levetiracetam saliva and serum concentrations (r^2^ = 0.8428–0.93) [20,77,79]. Grim et al. reported contrasting results: 40% lower levetiracetam concentrations in oral fluid than in serum [76]. The discrepancy is believed to be due to the different sampling and assay procedures [77].

### 4.12. Oxcarbazepine

Oxcarbazepine is licensed for monotherapy and for the adjunctive treatment of partial seizures, as well as in the treatment of bipolar disorder and trigeminal neuralgia [17]. Its metabolite is mono-hydroxycarbazepine (MHD or 10-hydroxycarbazepine or 10-hydroxy-10,11-dihydrocarbazepine), which has a plasma protein binding of 40%, a concentration that predicts good penetration into the oral fluid [16,17]. The AEDs that enhance its metabolism, leading to a 15–35% reduction in MHD plasma levels, are carbamazepine, lacosamide, phenobarbital and phenytoin. However, the AEDs that decrease its plasma concentrations by 11% and 20% are viloxazine and, respectively, verapamil [17].

Oxcarbazepine is pharmacologically active but is often at a very low, undetectable concentrations. Therefore, the levels of its metabolite, MHD, are routinely monitored [17].

Unstimulated oral fluid/serum MHD levels’ correlation values range from r^2^ = 0.91 to 0.96 [16,80,81]. Stimulated saliva flow can cause a decrease in saliva MHD levels, so that saliva MHD approaches the range of unbound MHD concentrations in serum or plasma. However, increasing the oral fluid flow disrupts the normal correlation between saliva and serum MHD concentrations [81]. Therefore, there is a wide variation between the correlation values when stimulated saliva is collected, with values of 0.21–0.68 [16,82,83,84]. The time of the fluid collection is another aspect of interest. Miles et al. concluded that unstimulated saliva/plasma ratios correlated well in the 8–72 h window after oral oxcarbazepine administration, but not earlier than 8 h. Therefore, the authors report that saliva is a suitable matrix for MHD TDM and recommend that oral fluid collection should be avoided within 8 h of the last orally administered dose [81].

### 4.13. Perampanel

Perampanel is prescribed in the adjunctive treatment of partial-onset seizures and of primary generalized tonic–clonic seizures in patients with idiopathic generalized epilepsy [17]. Perampanel has potentially significant drug–drug interactions; carbamazepine, oxcarbazepine, phenytoin and topiramate can decrease perampanel plasma concentrations by enhancing its metabolism, whilst ketoconazole can increase perampanel plasma concentrations by inhibiting its metabolism [16,17]. Although perampanel TDM is not routinely recommended, when patients are taking concomitant medications monitoring is suggested, due to the various possible drug interactions [16].

Perampanel is 98% bound to plasma proteins. To date, there are no studies that recommend saliva as a matrix for perampanel TDM [16,17].

### 4.14. Phenobarbital

Phenobarbital is prescribed in the treatment of all forms of epilepsy, except absence seizures. Other indications include acute convulsive episodes and status epilepticus, Lennox–Gastaut syndrome, myoclonic seizures, neonatal seizures and in the prophylaxis of febrile seizures [17]. Phenobarbital’s metabolism is inhibited and plasma concentrations are increased by acetazolamide, felbamate, methsuximide, oxcarbazepine, phenytoin, retigabine, rufinamide, stiripentol, sulthiame, chloramphenicol, propoxyphene and valproic acid. On the other hand, phenobarbital’s metabolism is enhanced and plasma concentrations are lowered by dicoumarol, thioridazone and troleandomycin [16,17]. The significant amount of possible drug–drug interactions and variable dose-concentration relationships recommend phenobarbital TDM [16].

Phenobarbital is 48–55% bound to plasma proteins and it distributes into saliva [16,17]. Since approximately 50% of phenobarbital is charged in blood and oral fluid at physiological pH, its penetration in oral fluid is unreliable [16]. Conclusively, in the literature, oral fluid/blood ratios for phenobarbital have been reported to range from r^2^ = 0.20 to r^2^ = 0.52 for total phenobarbital, and from r^2^ = 0.63 to r^2^ = 0.68 for free phenobarbital. Oral fluid phenobarbital concentrations correlate with blood phenobarbital concentrations at values of r^2^ = 0.64–0.98 for total phenobarbital and r^2^ = 0.64–0.99 for free phenobarbital [15,16,63]. Carvalho et al., adapting cards that are commonly applied in dried blood spots to oral fluid samples and using LC coupled to a DAD, determined phenobarbital at r^2^ = 0.998 ± 0.001, with a mean recovery of 50.6 ± 6.5 from dried saliva spots [59]. Therefore, saliva sampling is concluded to be a suitable matrix for phenobarbital TDM. 

### 4.15. Phenytoin

Phenytoin is prescribed in the treatment of tonic–clonic seizures and focal seizures, as well as trigeminal neuralgia and seizures that occur during or following severe head injury and/or neurosurgery [17]. Phenytoin is subjected to more drug–drug interactions than any other AED, with a long list of AEDs that can increase its blood concentrations (acetazolamide, clobazam, eslicarbazepine acetate, felbamate, methsuximide, oxcarbazepine, rufinamide, stiripentol, sulthiame and topiramate). Carbamazepine, phenobarbital and valproic acid can either increase or decrease its blood concentrations. Non-epilepsy drugs that can affect phenytoin metabolism are numerous, as well [16,17,52]. Phenytoin is 92% bound to plasma protein, exhibiting non-linear plasma pharmacokinetics that occur at different doses for different patients. Given all these facts, and phenytoin’s narrow therapeutic window, TDM is strongly recommended [16,17].

Phenytoin’s distribution into saliva reflects the non-protein bound levels in plasma, with correlation coefficients of r^2^ = 0.92–0.99 for oral fluid/total phenytoin and r^2^ = 0.98–0.99 for oral fluid/free phenytoin [15,16,17]. Therefore, mean saliva to blood concentration ratios of total phenytoin vary from 0.09 to 0.13, whilst for free phenytoin the ratios vary from 0.99 to 1.06 [15,16]. Patrick et al., in their review study, noted that there are three main considerations when sampling saliva for phenytoin TDM: the saliva flow rate, the timing of the sampling and concomitant drug use. Apart from the aforementioned possible drug–drug interactions, the authors concluded that unstimulated saliva samples should be collected due to the fact that higher phenytoin concentrations have been found in unstimulated samples. Moreover, the sampling should be performed more than 4 h after the last phenytoin dose was administered in order to avoid any drug residue in the oral fluid that could alter the concentrations [16]. Several other studies confirmed literature values—the correlation of free plasma phenytoin levels (approximately 10% of total plasmatic values) and saliva phenytoin levels was r^2^ = 0.82–0.998 [59,61].

### 4.16. Pregabalin

Pregabalin is prescribed in the treatment of partial seizures, in anxiety disorders, panic disorder and for peripheral and central neuropathic pain. Pregabalin is not bound to plasma proteins and it is not metabolized. Gabapentin and phenytoin can decrease pregabalin plasma concentrations. To date, it is not known if pregabalin is secreted into saliva [15,17].

### 4.17. Primidone

Primidone is prescribed to treat generalized tonic–clonic, Jacksonian, psychomotor and focal seizures, as well as myoclonic jerks, essential tremor and akinetic attacks [17]. Primidone is 33% bound to plasma proteins [19,51]. Acetazolamide, carbamazepine and phenytoin can decrease plasma primidone concentrations, whilst clobazam, ethosuximide and stiripentol can increase those concentrations [17,52]. Primidone produces two pharmacologically active metabolites, phenobarbital and phenyl-ethyl-malondiamide, that are responsible for most of the drugs’ actions [17]. Therefore, saliva TDM for both primidone and phenobarbital is recommended, especially since blood primidone concentrations are correlated with saliva concentrations: r^2^ = 0.71–0.98 [15,16,17].

### 4.18. Rufinamide

Rufinamide is prescribed to treat seizures associated with Lennox–Gastaut syndrome, but also to treat partial seizures, epileptic spasms, myoclonic-astatic epilepsy and status epilepticus [17]. Carbamazepine, methsuximide, oxcarbazepine, phenobarbital, phenytoin, primidone and vigabatrin can induce and inhibit rufinamide’s metabolism, enhancing its elimination. Valproic acid increases rufinamide’s plasma concentrations [17,52,85,86]. Rufinamide is 28% bound to plasma proteins, with the saliva levels reflecting the non-protein bound plasma concentrations [15,17]. Franco et al. determined a correlation coefficient between saliva and plasma of r^2^ = 0.78, while stating that the rufinamide concentrations in saliva were moderately lower than those in plasma, with a mean saliva to plasma ratio of 0.7 ± 0.2 [85]. Mazzucchelli et al. reported a mean saliva to plasma concentration ratio of 0.66, a value that confirms that salivary rufinamide concentrations reflect the unbound drug concentrations in plasma [87]. Therefore, saliva is a suitable matrix for rufinamide TDM.

### 4.19. Topiramate

Topiramate is prescribed in the treatment of generalized tonic–clonic seizures and partial seizures, as well as for seizures associated with Lennox–Gastaut syndrome and in migraines [17]. AEDs such as carbamazepine, eslicarbazepine acetate, methsuximide, oxcarbazepine, phenobarbital, phenytoin, primidone and valproic acid lower topiramate plasma concentrations, whilst non-AEDs such as diltiazem, hydrochlorothiazide, lithium, metformin, propranolol, posaconazole and sumatriptan increase topiramate’s plasma concentrations [17,52].

Topiramate is 20% bound to plasma proteins and it is secreted into oral fluid, with a prediction for strong correlations between total plasma and saliva levels r^2^ = 0.92–0.98 [15,17,51,88]. As previously stated, consideration should be given to the time of the saliva sampling, regarding the last administered dose. Miles et al. collected unstimulated oral fluid samples > 3 h after the patients received their last topiramate dose [88]. Conclusively, saliva TDM is a good option for monitoring topiramate levels, when the time of sampling is being considered.

### 4.20. Valproic Acid (Valproate)

Valproic acid is prescribed in the treatment of any form of epilepsy in patients of any age, as well as in several seizure disorders (such as febrile seizures, infantile spasms, juvenile myoclonic epilepsy, Lennox–Gastaut syndrome, neonatal seizures, etc.) and non-epilepsy conditions (such as bipolar depression, psychosis or schizophrenia) [17]. Valproate plasma concentration is decreased by AEDs (such as carbamazepine, eslicarbazepine acetate, ethosuximide, lamotrigine, methsuximide, phenobarbital, phenytoin, primidone, tiagabine and topiramate) and by non-AEDs (such as amikacin, cisplatin, diflunisal, doripenem, efavirenz, ertapenem, imipenem, meropenem, methotrexate, naproxen, oral contraceptives, panipenam, rifampicin and ritonavir). On the other hand, valproate’s plasma concentration is increased by AEDs such as clobazam, felbamate and stiripentol, and, respectively, by non-AEDs such as bupropion, chlorpromazine, cimetidine, erythromycin, guanfacine, isoniazid, lithium, sertraline and verapamil [17,52].

Valproic acid’s protein-bound plasma level is concentration dependent, with variations from 74% to 93% [17,51]. Pastalos et al. suggested that saliva is not a useful matrix for valproic acid TDM due to the fact that the distribution of valproate in saliva is reported to be erratic [15,17]. Patrick et al. also predicted poor and inconsistent valproic acid penetration into saliva [16]. Saliva stimulation does not enhance the recovery of valproate in saliva and it does not improve the correlation between its salivary and serum-free concentrations [61]. Nevertheless, Dwivedi et al. reported a significant correlation (r^2^ = 0.36, *p* < 0.004), with a mean ratio of saliva to serum-free concentration of 0.68 ± 1.29% [89]. Another study by Tonic-Ribarska et al. studied the determination of valproic acid from unstimulated saliva samples, reporting a mean recovery of 99.4% with a concentration coefficient for the calibration function for valproate of r^2^ = 0.9989 [90]. Al Za’abi et al. also reported a good linear relationship between the salivary and the serum-free valproic acid, with a correlation coefficient of r^2^ = 0.70 (*p* < 0.04) [61]. More studies are required in order to make saliva an appropriate matrix for valproic acid TDM. 

The data collected in this narrative review are summarized in Table 1. For each AED, the published studies found are noted (with regard to authors, journal and year of publication). For each AED in particular, the biofluid which saliva AED levels were compared to is noted in the “Biofluid 1” column. Additionally, the method of determination of the AED levels from each biofluid and the correlations between the two measurements are noted. The AEDs included in this table are the AEDs that show promise in salivary biomarker detection.

## 5. Discussion

AEDs are numerous and diverse, with different mechanisms of action, and choosing the right anti-epileptic for a patient is based on numerous factors such as the seizure type, the potential for drug interactions and the associated comorbidities. The initial response of a patient to a prescribed AED and the monitoring of the dosages is traditionally performed through blood sampling TDM. This monitoring is important because of the clinically relevant differences that exist among similarly active AEDs. Moreover, their possible interactions can lead to both beneficial and/or undesirable effects [14]. Furthermore, the importance of AED and TDM also comes from the statement that about 30% of patients are refractory or drug resistant to AEDs [20].

Numerous AEDs have great potential to be routinely determined through saliva sampling, especially clobazam, clonazepam, diazepam, midazolam, carbamazepine, gabapentin, lacosamide, lamotrigine, levetiracetam, oxcarbazepine, phenobarbital, phenytoin, primidone, rufinamide, topiramate and valproic acid. Saliva TDM would greatly facilitate AED administration for practitioners through its rapidity, ease and a better avoidance of possible side effects. Future research should be focused in order to study and confirm the correlations between these AEDs’ blood and saliva levels. Additionally, more research is needed to create a basis for saliva TDM for all the other generally prescribed AEDs.

There is usually a constant proportion between the non-protein-bound AED concentration and the protein-bound. In some cases, when protein binding is influenced by various pathologies (such as renal or hepatic diseases), the free non-protein bound dictates the therapeutic outcome and serves as a clinical guideline for dose management. Therefore, traditionally, the free pharmacologically active concentrations of the component are measured through blood withdrawals in order to adapt patients’ doses [15,17]. The blood withdrawals should be performed at the moment when the AED reaches its plasmatic peak in order to monitor the effects. The AED oral dose might be constant, whilst the plasma levels, however, may be low, which can cause seizures to appear. In these cases, it is essential to perform TDM correctly [91]. Saliva TDM of AEDs can be carried out, however, as an alternative assessment sampling technique, with knowing the precise timing of the drug’s blood:saliva equilibrium for each patient [16]. The majority of AEDs, because of their lipophilic properties, cross the blood–brain barrier and can be determined from saliva [16]. Further research is needed in order to determine the right moment for sampling—especially when switching the medications, when multiple AEDs are prescribed or in pharmacologically resistant patients [17,20,21].

With regard to the ideal biofluid with which to compare saliva AEDs levels, further research is needed. Many of the described studies used in vitro enhanced biofluids to establish correlations, while other studies tested AEDs levels in both healthy and epileptic patients. The metabolic response between subjects with epilepsy and healthy subjects under AEDs treatment is different. Another aspect is that, in general, plasma and serum levels are comparable. Serum is the liquid that remains after the blood has clotted, while plasma is the liquid that remains when clotting is prevented with the addition of an anticoagulant. However, the use of said anticoagulant can impact the plasma TDM, and the results may vary from one study to another [92,93]. All these differences between the biofluids require enhancement and a predictability of the TDM process. Saliva TDM is a valid option in order to monitor AEDs levels. Further research regarding better-established protocols is, however, needed.

The comparisons between AEDs levels between saliva and another biofluid (e.g., blood or urine) have not taken into consideration modifications due to pH, biofluid density, composition or due to any other pathologies or concomitant drug intakes (drugs prescribed for pathologies other than epilepsy). Additionally, AED monitoring through saliva is a practice that is gaining popularity due to several advantages. Its ease in collecting and storing even multiple samples at a time and the lack of invasiveness might result in it being the future matrix of choice for AED TDM [1,16,17].

There are a few disadvantages associated with saliva TDM, such as the modifications of the oral fluid flow rate, consistency and collected amounts that vary from one patient to another. Moreover, there are difficulties in saliva sampling in certain populations with xerostomia or with critical illnesses. Other drawbacks include the contamination of saliva samples with food, with various periodontal and dental caries microorganisms and even with blood from periodontal pockets [1,17]. There is also a very short period of time available for drug detection in saliva: about 12–24 h after consumption [35].

The benefits of using saliva as AED TDM, however, outweigh the drawbacks. Therefore, there is an increased demand for further research in order to improve the detection and the surveillance of biomarkers. The techniques for biomarker determination should be low-cost and simple to use and to integrate in healthcare centers. Moreover, further research should also be aimed at the improvement of electrochemical sensors in order to more selectively and concomitantly determine multiple types of biomarkers from oral fluid samples. The saliva TDM of AEDs should be determined correctly, quickly and accurately with a universal test that can correlate the oral fluid drug levels to plasma levels.

Overall, saliva analysis is a promising way to monitor numerous biomarkers, having significant potential to be used when fast, efficient and specific determinations are needed, hence its applicability in the future in emergency rooms or even schools, workplaces or roadside testing with law enforcement officers [1]. As a general future perspective, monitoring salivary biomarkers has great potential in being a selective means of analysis in numerous medical and legal fields. 

## 6. Conclusions

In various pathologies, the TDM of the drugs prescribed as treatment is required so that the doses can be monitored and updated if needed. In several cases, saliva has become, instead of blood or plasma, the matrix of choice for testing. All that being said, there is a need for further research regarding the sensitivity of the qualitative and quantitative determination of saliva biomarkers from oral fluid samples. With the proper adaptations and the right analytical methods, saliva TDM has great potential to be used and perfected—notably in long-term treatments that need constant monitorization and updating.

## Figures and Tables

**Table 1:** Correlations in the determination of salivary levels of individual AEDs.

AED (and Their Metabolites)	Authors	Publication	Year	Biofluid 1	Biofluid 2	Determination Method	Correlation/Corresponding Results * Between the Biofluids
Acetazolamide	Wallace et al. [23]	*J Pharm Sci*	1977	Enhanced Plasma	Enhanced Saliva	GLC	0.99
	Hartley et al. [24]	*J Chromatogr.*	1986	Enhanced Plasma	Enhanced Saliva	HPLC	0.985
Clobazam and N-desmethyl clobazam	Gorodischer et al. [30]	*Ther Drug Monit.*	1997	Plasma	Saliva	GC	0.9 (clobazam), 0.93 (N-desmethyl clobazam)
	Bardy et al. [31]	*Brain Dev.*	1991	Serum	Saliva	HPLC and enzyme multiplied immunoassay technique	0.9 (clobazam), 0.93 (N-desmethyl clobazam)
Clonazepam and 7-acetamidoclonazepam	Moore et al. [33]	*J Anal Toxicol*	2007	Enhanced Artificial Saliva	-	LC-MS/MS	0.9991
	Bakke et al. [29]	*J Anal Toxicol.*	2019	Blood	Saliva	UHPLC-MS/MS	71% *
	Desharnais et al. [34]	*Forensic Sci Int*	2020	Enhanced Saliva	-	LC-MS	-
	Uddin et al. [27]	*J Sep Sci.*	2008	Enhanced Plasma	Enhanced Saliva	HPLC	0.999
	Concheiro et al. [35]	*Anal Bioanal Chem*	2008	Enhanced Saliva	-	LC-MS	0.99
	Øiestad et al. [36]	*Clin Chem*	2007	Enhanced Saliva	-	LC-MS	0.993
	Gunnar et al. [37]	*J Mass Spectrom JMS*	2005	Enhanced Saliva	-	GC-MS	0.992
Diazepam, nordiazepam and o N-desmethyldiazepam, 3- OH-diazepam, temazepam and oxazepam	Hallstrom et al. [38]	*Br J Clin Pharmacol*	1980	Plasma	Saliva	GC	0.89 (diazepam), 0.81 (nordiazepam)
	Moore et al. [33]	*J Anal Toxicol.*	2007	Enhanced Artificial Saliva	-	LC-MS/MS	0.9996 (diazepam)
	Gunnar et al. [37]	*J Mass Spectrom JMS*	2005	Enhanced saliva	-	GC-MS	1.000 (diazepam, 0.999 (temazepam), 0.998 (nordiazepam and oxazepam)
	Bakke et al. [29]	*J Anal Toxicol.*	2019	Blood	Saliva	UHPLC-MS/MS	96.2% * (diazepam), 100% * (N-desmethyldiazepam), 88.9% * (oxazepam)
	Vindenes et al. [22]	*J Anal Toxicol.*	2011	Urine	Saliva	LC-MS	89% * (N-desmethyldiazepam), 75% * (3-OH-diazepam), 68% * (oxazepam)
	Gjerde et al. [39]	*J Anal Toxicol.*	2010	Blood	Saliva	HPLC-MS/MS	0.61 (diazepam), 0.95 (nordiazepam)
Midazolam and 1-hydroxymidazolam and 4-hydroxymidazolam	Link et al. [41]	*Rapid Commun Mass Spectrom*	2007	Enhanced Plasma	Enhanced Saliva	LC-MS	0.9991 (midazolam), 0.9978 (1-hydroxymidazolam), 0.9986 (4-hydroxymidazolam)
	Link et al. [42]	*Br J Clin Pharmacol.*	2008	Plasma	Saliva	LC-MS/MS	0.864 (midazolam)
	Moore et al. [33]	*J Anal Toxicol.*	2007	Enhanced Artificial Saliva	-	LC-MS/MS	0.996 (midazolam)
	Gunnar et al. [37]	*J Anal Toxicol.*	2007	Enhanced Artificial Saliva	-	LC-MS/MS	0.997 (midazolam)
	Donzelli et al. [43]	*Clin Pharmacokinet.*	2014	Plasma	Saliva	HPLC-MS/MS	0.886–0.959 (midazolam)
Brivaracetam	Rolan et al. [50]	*Br J Clin Pharmacol.*	2008	Plasma	Saliva	LC-MS	0.97
Carbamazepine and carbamazepine-10,11-epoxide	Vasudev et al. [58]	*Neurol India*	2002	Serum	Saliva	HPLC	0.659
	Dordevic et al. [53]	*Vojnosanit Pregl.*	2009	Serum	Saliva	HPLC	0.9481
	Al Za’abi et al. [61]	*Acta Neurol Belg.*	2003	Serum	Saliva	Fluorescence polarization immunoassay	0.99
	Djordjevic et al. [55]	*Vojnosanit Pregl.*	2012	Serum	Saliva	HPLC-UV	0.9117
	Carona et al. [20]	*J Pharm Biomed Anal.*	2021	Plasma	Saliva	HPLC	0.8299 (carbamazepine) 0.9291 (carbamazepine-10,11-epoxide)
	Dziurkowska and Wesolowski [62]	*Mol Basel Switz.*	2019	Saliva	-	UHPLC-MS/MS-DAD	>0.99 (carbamazepine-10,11-epoxide)
	Carvalho et al. [59]	*J Anal Toxicol.*	2019	Dried Enhanced Saliva Spots	-	HPLC-DAD	0.998
	Dwivedi et al. [63]	*Int J Neurosci.*	2016	Serum	Saliva	HPLC	0.6614
	Chen et al. [60]	*Biomed Opt Express.*	2021	Enhanced Saliva	-	SERS	0.9663–0.9753
Ethosuximide	Patrick et al. [16]	*Ther Drug Monit.*	2013	Blood	Saliva	GC	0.74–0.99
Gabapentin	Pujadas et al. [54]	*J Pharm Biomed Anal.*	2007	Enhanced Saliva	-	GC-MS	0.9903
	Berry et al. [66]	*Seizure*	2003	Plasma	Saliva	HPLC	0.9491
Lacosamide	Carona et al. [20]	*J Pharm Biomed Anal.*	2021	Plasma	Saliva	HPLC	0.9912
	Greenaway et al. [68]	*Epilepsia*	2011	Serum	Saliva	HPLC	0.842
	Brandt et al. [67]	*Epilepsia*	2018	Serum	Saliva	Unstated	0.578–0.671
	Cawello et al. [69]	*Epilepsia*	2013	Plasma	Saliva	HPLC-MS	0.9496–0.9577
	Patrick et al. [16]	*Ther Drug Monit.*	2013	Blood	Saliva	HPLC	0.84–0.98
Lamotrigine	Tsiropoulos et al. [71]	*Ther Drug Monit.*	2000	Serum	Saliva	HPLC	0.85 (unstimulated) 0.94 (stimulated saliva)
	Malone et al. [72]	*J Clin Neurosci Off J Neurosurg Soc Australas.*	2006	Plasma	Saliva	HPLC	0.9841
	Incecayir et al. [73]	*Arzneimittelforschung*	2007	Plasma	Saliva	HPLC	0.677
	Mallayasamy et al. [74]	*Arzneimittelforschung*	2010	Plasma	Saliva	HPLC	0.6832
	Ryan et al. [75]	*Pharmacotherapy*	2003	Serum	Saliva	HPLC	0.905, 0.940
	Patrick et al. [16]	*Ther Drug Monit.*	2013	Blood	Saliva	HPLC	0.95
Levetiracetam	Lins et al. [78]	*Int J Clin Pharmacol Ther.*	2007	Plasma	Saliva	Unstated	0.88
	Grim et al. [76]	*Ther Drug Monit.*	2003	Serum	Saliva	HPLC	0.87, 0.86
	Carona et al. [20]	*J Pharm Biomed Anal.*	2021	Plasma	Saliva	HPLC	0.8428
	Mecarelli et al. [77]	*Ther Drug Monit.*	2007	Serum	Saliva	GC	0.9
	Hamdan et al. [79]	*J Anal Methods Chem.*	2017	Plasma	Saliva	HPLC	0.9
	Patrick et al. [16]	*Ther Drug Monit.*	2013	Blood	Saliva	HPLC	0.91 (unstimulated), 0.87 (stimulated saliva)
Oxcarbazepine and mono-hydroxycarbazepine	Li et al. [80]	*Ther Drug Monit.*	2016	Plasma	Saliva	HPLC	0.908
	Miles et al. [81]	*Ther Drug Monit.*	2004	Serum	Saliva	HPLC	0.941
	Klitgaard et al. [82]	*Eur J Clin Pharmacol.*	1986	Plasma	Saliva	Equilibrium dialysis and an ultrafiltration technique	0.75
	Kristensen et al. [84]	*Acta Neurol Scand.*	1983	Serum	Saliva	HPLC	0.914
	Patrick et al. [16]	*Ther Drug Monit.*	2013	Blood	Saliva	HPLC	0.91–0.98
Phenobarbital	Dwivedi et al. [63]	*Int J Neurosci.*	2016	Serum	Saliva	HPLC	0.4257
	Carvalho et al. [59]	*J Anal Toxicol.*	2019	Dried Enhanced Saliva Spots	-	HPLC-DAD	0.998
	Patsalos and Berry [15]	*Ther Drug Monit.*	2013	Blood	Saliva	Unstated	0.91
	Patrick et al. [16]	*Ther Drug Monit.*	2013	Blood	Saliva	HPLC	0.91–0.94
Phenytoin	Carvalho et al. [59]	*J Anal Toxicol.*	2019	Dried Enhanced Saliva Spots	-	HPLC-DAD	0.998
	Al Za’abi et al. [61]	*Acta Neurol Belg.*	2003	Serum	Saliva	Fluorescence polarization immunoassay	0.98
	Patrick et al. [16]	*Ther Drug Monit.*	2013	Blood	Saliva	HPLC	0.92–0.99
	Patsalos and Berry [15]	*Ther Drug Monit.*	2013	Blood	Saliva	Unstated	0.85–0.99
Primidone	Patrick et al. [16]	*Ther Drug Monit.*	2013	Blood	Saliva	HPLC	0.71–0.98
	Patsalos and Berry [15]	*Ther Drug Monit.*	2013	Blood	Saliva	Unstated	0.71–0.97
Rufinamide	Franco et al. [85]	*Epilepsia*	2020	Plasma	Saliva	HPLC-UV	0.78
	Mazzucchelli et al. [87]	*Anal Bioanal Chem*	2011	Plasma	Saliva	HPLC-UV	0.99
Topiramate	Miles et al. [88]	*Pediatr Neurol*	2003	Serum	Saliva	Fluorescence polarization immunoassay	0.97
	Patsalos and Berry [15]	*Ther Drug Monit.*	2013	Blood	Saliva	Unstated	0.97
Valproic acid	Al Za’abi et al. [61]	*Acta Neurol Belg.*	2003	Serum	Saliva	Fluorescence polarization immunoassay	0.7
	Dwivedi et al. [89]	*Seizure*	2015	Serum	Saliva	HPLC	0.13
	Tonic-Ribarska et al. [90]	*Acta Pharm Zagreb Croat.*	2012	Saliva	-	HPLC	0.9989

* corresponding results (%) were noted in articles that did not state a correlation coefficient. GC = gas chromatography, GLC = gas–liquid chromatography, HPLC = high-performance liquid chromatography, HPLC-DAD = high-performance liquid chromatography with diode-array detection, HPLC-UV= high-performance liquid chromatography with ultraviolet spectroscopy, LC-MS = liquid chromatography mass-spectrometry, LC-MS/MS = liquid chromatography tandem mass-spectrometry, SERS = surface-enhanced Raman spectroscopy, UHPLC-MS/MS = ultra-high performance liquid chromatography.

## Data Availability

Not applicable.
